# Supplementary material for: Designer liquid-liquid interfaces made from transient double emulsions
Source: Nat Commun. 2018 Nov 12;9:4763. doi: 10.1038/s41467-018-07272-0 (PMC6232135; doi:10.1038/s41467-018-07272-0)
Supplement: Supplementary file 1 — Supplementary Information [file 41467_2018_7272_MOESM1_ESM.pdf]

Supplementary Information

Designer liquid-liquid interfaces made from out-of-equilibrium double emulsions.

Dockx and Geisel et al.

Supplementary Note 1: Predicting the surface coverage

The desired surface coverage $\Phi_{\mathrm{s,def}}$ for a spherical droplet of size d and particles of size d_{p} and having a contact angle θ can be calculated as followed:

$$\Phi_{\mathrm{s}} = \frac{N_{\mathrm{p}}(d_{\mathrm{p}} \sin\theta)^2}{4d_{\mathrm{d}}^2}, \tag{1}$$

with N_{p} the number of adsorbed particles at the interface of the droplet. N_{p} can be determined from the mass balance as is assumed that all the particles go to the interface. The mass of particles in one droplet $W_{\mathrm{p,1droplet}}$ depends on the flow rates of the middle phase Q_{M}, the inner phase Q_{I} and the weight fraction of particles in the middle phase w_{p}. When the middle phase dissolves into the outer phase the mass is:

$$W_{\mathrm{p,1droplet}} = \frac{\pi d_{\mathrm{d}}^3}{6}\frac{Q_{\mathrm{M}}}{Q_{\mathrm{I}}}\rho_{\mathrm{M,p}} w_{\mathrm{p}}, \tag{2}$$

For the middle phase dissolving into the inner one, the droplet volume changes and the equation becomes:

$$W_{\mathrm{p,1droplet}} = \frac{\pi d_{\mathrm{d}}^{3}}{6} \frac{Q_{\mathrm{M}}}{(Q_{\mathrm{M}} + Q_{\mathrm{I}})} \rho_{\mathrm{M,p}} w_{\mathrm{p}}, \tag{3}$$

The number of particles can then be calculated by dividing the total mass of particles by the mass of one particle so that the surface coverage is equal to:

$$\Phi_{\mathrm{s,def}} = \frac{1}{4} \frac{d_{\mathrm{d}} \sin^{2}\theta}{d_{\mathrm{p}}} \frac{Q_{\mathrm{M}}}{Q_{\mathrm{I}}} \frac{\rho_{\mathrm{M,p}}}{\rho_{\mathrm{p}}} w_{\mathrm{p}} \tag{4}$$

When the middle phase dissolves into the outer phase. In the other case the nominator changes to $(Q_{\mathrm{M}} + Q_{\mathrm{I}})$.

For droplets in the channels, which are deformed into pancakes, the droplet area increases and different geometrical formula are needed.

Supplementary Note 2: Stability of the produced droplets

Supplementary figure 1.b represents the surface coverage of droplet with a diameter of 430 μm and a height of 250 μm covered with spherical 600 nm silica particles for different particle concentrations and wettabilities using Supplementary Equation 4 and using the phase diagram in Supplementary Fig. 1.a to get the

equilibrium concentrations. $Q_{\mathrm{M}}/Q_{\mathrm{I}}$ was equal to one. From Supplementary Fig. 1.g and Supplementary Fig. 1.h, it can be seen that the particle concentrations 12 $\mathrm{mg\,mL^{-1}}$ and 25 $\mathrm{mg\,mL^{-1}}$ correspond, respectively, to the limit of complete surface coverage and excess-of-particles regime. For a concentration of 12 $\mathrm{mg\,mL^{-1}}$, it was still possible to collect stable droplets and, denser layer was formed.

Pickering droplets were created with the middle phase containing 12 $\mathrm{mg\,mL^{-1}}$ or 25 $\mathrm{mg\,mL^{-1}}$ of ODTMS treated fluorescent 600 nm silica particles and using flow rates $Q_{\mathrm{O}} = 7000\ \mu\mathrm{L\,h^{-1}}$ and $Q_{\mathrm{M}} = Q_{\mathrm{I}} = 500\ \mu\mathrm{L\,h^{-1}}$. Supplementary Fig. 2.a shows the droplet stability after 4 weeks. A cellular network is formed upon evaporation of the outer phase, as shown in Supplementary Fig. 2.b. Supplementary Fig. 2.c shows what happens when the aged droplets are squeezed between two coverslips and fractures are observed, showing the viscoplastic nature of the interface. Supplementary Fig. 2.d and Supplementary Fig. 2.e illustrate that the stability of the non-coalescenced and arrested droplets produced with a particle concentration of 12 mg/mL is at least 6 months. The particles in the continuous phase come from pipetting the emulsion onto a coverslip, which destroys multiple droplets. Moreover, these figures illustrate the excellent control over the surface

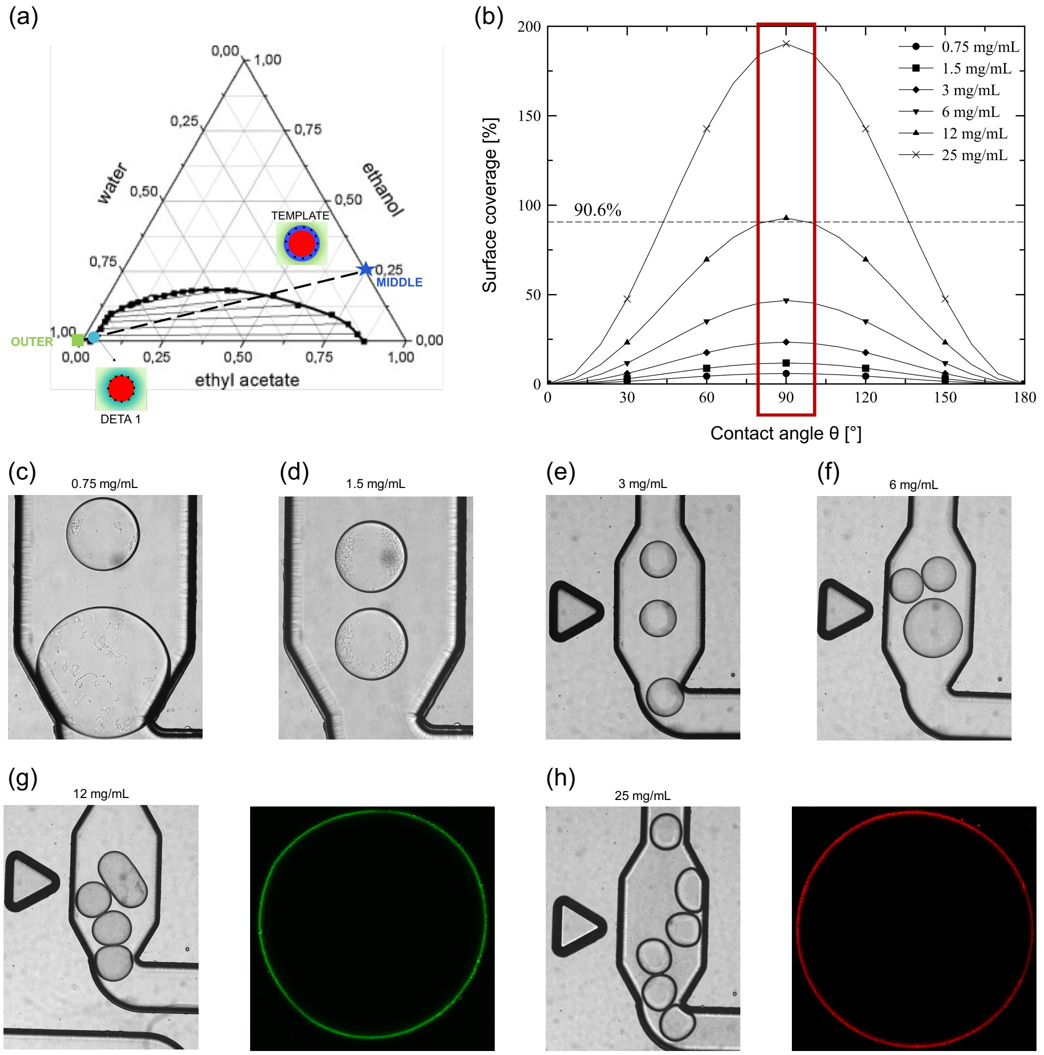

Supplementary Figure 1: **a** Ternary diagram of ethyl acetate, ethanol and water at 293.15 K in mole fraction with the location of the middle and outer phase. The mixing point is located on the left of the binodal region. **b** Theoretical surface coverage calculated for the different particle concentrations and wettabilities. **c-h** Brightfield images of the droplets at the outlet of the microfluidic device for the corresponding particle concentrations. Confocal images of the droplet cross-section are also shown when it was possible to collect stable droplets (width image = 246.27 μm).

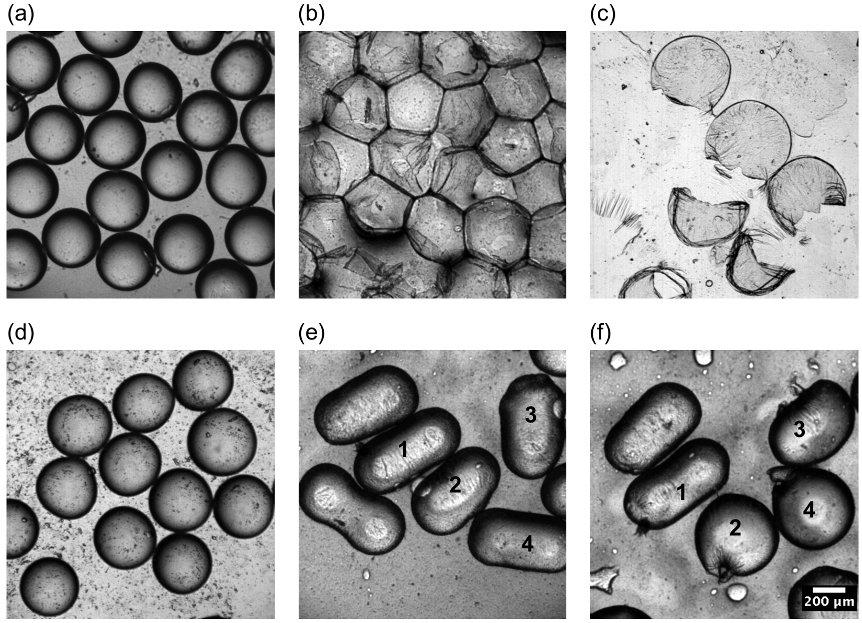

Supplementary Figure 2: Emulsion after 4 weeks, produced with 25 $\mathbf{mg\,mL^{-1}}$: **a** Non-arrested droplets, **b** After evaporation of the outer phase, **c** Droplets fracture by squeezing them between two coverslips. Emulsion after 6 months, produced with 12 $\mathrm{mg\,mL^{-1}}$: **d** Non-arrested droplets, **e** Arrested droplets, **f** Same sample as in **e** after manipulation with a sharp needle.

coverage as Supplementary Fig. 2.d and Supplementary Fig. 2.e come for two different experiments with slightly different particle concentrations, namely 12.1 $\mathrm{mg\,mL^{-1}}$ and 12.0 $\mathrm{mg\,mL^{-1}}$, respectively. The nonspherical structures clearly confirm the presence of an interfacial particle network that can bear stresses without massive particle expulsion.

Supplementary Note 3: Confocal imaging

To collect droplets from the microfluidic device, they have to be stabilized. This was only the case for droplets completely covered with a close-packed particle layer in the experiments performed in this thesis work as illustrated by Supplementary Fig. 3.b in the main manuscript. Such emulsion droplets were stable for a long time, at least 10 months. Phase inversion of the emulsion was observed for hydrophobic particles, while a stable emulsion could be collected for particles with favourable wetting properties. However, some droplets of the emulsion broke after one day as a small amount of particles were observed at the bottom of the vial. The microscopic images were taken while collecting droplets with favourable wetting properties in a petridish.

Supplementary Figure 2.b presents a z-scan of an O/W droplet that was produced with a surface coverage close to maximum packing. As outer phase water with 50% glycerol was used. As inner and middle phase we used a mixture of hexadecane and 1-decanol (90.5%, 9.5%) and hexadecane, 1 butanol, 1-decanol (2.9%, 89.1%,8.0%) as inner phase.The images illustrate the presence of a close-

packed crystalline shell at the droplets and no particles inside the droplet. Confocal images were obtained for of an O/W droplet of 138 $\pm$ 7 μm covered with 0.5% ODTMS treated 630 silica particles at different heights. A satellite droplet is observed at h = 19.1 μm. The experiment was performed with a particle concentration of 14.8 wt% and flow rates $Q_{\mathrm{O}} = 5100\ \mu\mathrm{L\,h^{-1}}$, $Q_{\mathrm{M}} = 100\ \mu\mathrm{L\,h^{-1}}$ and $Q_{\mathrm{I}} = 250\ \mu\mathrm{L\,h^{-1}}$.
