# Supplementary material for: Designer liquid-liquid interfaces made from transient double emulsions
Source: Nat Commun. 2018 Nov 12;9:4763. doi: 10.1038/s41467-018-07272-0 (PMC6232135; doi:10.1038/s41467-018-07272-0)
Supplement: Supplementary file 3 — Description of Additional Supplementary Files [file 41467_2018_7272_MOESM3_ESM.pdf]

Description of Additional Supplementary Files

File Name: Supplementary Movie 1
Description: Supplementary Movie 1 shows how droplets with a surface coverage of 85.5% were brought together in a diverging zone of the microfluidic chip. The droplets merge, but coalescence is clearly arrested and a snow man is created. When more droplets arise, highly controlled anisotropic supra-colloidal sausages are created.
